# Can composite digital monitoring biomarkers come of age? A framework for utilization

**DOI:** 10.1017/cts.2018.4

**Published:** 2018-04-23

**Authors:** Christopher Kovalchick, Rhea Sirkar, Oliver B. Regele, Lampros C. Kourtis, Marie Schiller, Howard Wolpert, Rhett G. Alden, Graham B. Jones, Justin M. Wright

**Affiliations:** 1 Eli Lilly Innovation Center, Cambridge, MA, USA; 2 Clinical & Translational Science Institute, Tufts University Medical Center, Boston, MA, USA

**Keywords:** Digital medicine, biomarkers, closed loop, sensors, IoT

## Abstract

**Introduction:**

The application of digital monitoring biomarkers in health, wellness and disease management is reviewed. Harnessing the near limitless capacity of these approaches in the managed healthcare continuum will benefit from a systems-based architecture which presents data quality, quantity, and ease of capture within a decision-making dashboard.

**Methods:**

A framework was developed which stratifies key components and advances the concept of contextualized biomarkers. The framework codifies how direct, indirect, composite, and contextualized composite data can drive innovation for the application of digital biomarkers in healthcare.

**Results:**

The de novo framework implies consideration of physiological, behavioral, and environmental factors in the context of biomarker capture and analysis. Application in disease and wellness is highlighted, and incorporation in clinical feedback loops and closed-loop systems is illustrated.

**Conclusions:**

The study of contextualized biomarkers has the potential to offer rich and insightful data for clinical decision making. Moreover, advancement of the field will benefit from innovation at the intersection of medicine, engineering, and science. Technological developments in this dynamic field will thus fuel its logical evolution guided by inputs from patients, physicians, healthcare providers, end-payors, actuarists, medical device manufacturers, and drug companies.

## Introduction and Current State

Incorporation of the term biomarker into medical parlance spans 4 decades and several of such have now become well known [1]. Prominent examples include HbA1c as a diagnostic marker for type II diabetes [2], prostate-specific antigen level for prostate cancer prognosis [3] and staging, and BRCA 1 and 2 for breast cancer genotyping [4]. Despite concerted efforts however (which have led to the discovery of extremely large and rapidly growing numbers of potential biomarkers) the translation and adoption of biomarkers as clinically validated surrogate endpoints of disease has moved at a relatively conservative pace [5]. Recent progress has been accelerated through availability of sensitive and rapid genomic, proteomic, and metabolomic interrogation tools [6], and as these analyses are embedded in clinical studies it is likely that new validated endpoints will be established. Leading the efforts to co-ordinate development of new biomarkers in the United States is the National Biomarker Development Alliance [7]. Established in 2014, its expressed mission is to create standards that can be used for evidence-based biomarker development, and their subsequent adoption to advance precision medicine [7]. Given the regulatory significance of biomarkers in clinical medicine, a joint working group composed of Food and Drug Administration (FDA) and National Institutes of Health representatives was formed to establish a framework including relevant nomenclature and recently issued guidelines [8]. Published late 2016, the guidelines (referred to as Biomarkers, Endpoints, and other Tools or BEST) are envisioned as a live/working document that can evolve as new developments are recommended. The BEST framework advocates for 7 independent categories viz. (1) susceptibility/risk biomarkers, (2) diagnostic biomarkers, (3) monitoring biomarkers, (4) prognostic biomarkers, (5) predictive biomarkers, (6) pharmacodynamic/response biomarkers, and (7) safety biomarkers. It is noted that depending on context of use, biomarkers from different categories may interchange and overlap. For example, diagnostic and prognostic biomarkers may be classified as monitoring biomarkers when they are measured serially.

The pursuit and development of clinically relevant biomarkers has also fueled growth of the companion diagnostics industry—a notable example being the CD340 biomarker used to identify patients for trastuzumab (Herceptin^®^), the first in class Dx/Rx combination for treatment of HER2-positive breast cancers [9]. Harnessing the power of such omic profiling techniques has resulted in considerable progress in the oncology field [10] and has impacted clinical trial stratification in other therapeutic areas including hypothyroid condition, cardiometabolic disease, as well as rare and orphan diseases [6, 11]. Although blood analysis can yield detailed and insightful diagnostic information, in the case of neurodegenerative disorders, routine analysis of cerebrospinal fluid for circulating biomarkers presents an obvious logistical barrier [12]. Compounding this problem is the fact that by the time many neurodegenerative diseases are diagnosed using conventional assessment methods, the disease may have advanced to a point where therapeutic options become limited. Accordingly, there is considerable interest in the application of noninvasive imaging techniques such as positron emission topography (PET) and functional magnetic resonance imaging to identify biomarkers of neurodegenerative disease based on metabolic and anatomic signatures, respectively. Given the potential impact of Alzheimer disease on the healthcare system and the relative low spend on this area relative to other chronic diseases such as cancer and rare diseases [13], such biomarkers are sorely needed, as the definitive marker remains assessment of amyloid plaque composition postmortem via autopsy [14]. Such biomarkers could also provide surrogate endpoints for the development of *chemopreventative* agents, allowing appropriate longitudinal studies to be designed. One such study, known as the Alzheimer’s disease neuroimaging initiative (ADNI) is currently underway, correlating changes in patients memory and functional capacity with anatomic and metabolic signatures derived from magnetic resonance imaging/PET imaging [15]. Though powerful, the high cost and limited availability of these imaging tools has fueled interest in methods able to identify and triage patients who are likely to benefit most. Numerous clinical studies are underway, including the Baltimore Longitudinal Study of Aging [16], which aims to correlate decline in cognitive and physical function with onset of neurodegenerative disease. Complimenting these efforts are myriad large scale trials which track patient health indicators using commoditized electronic devices and wearable technologies [17–21]. Initial results have been promising, suggesting the very real potential for use of noninvasive digital tools which might track healthy individuals as they progress to early onset diseased states [22, 23]. As such, the era of digital monitoring biomarkers (DMBs) is dawning, with the capacity to revolutionize aspects of the healthcare ecosystem if implemented appropriately [24–26]. Continual advances in technology development and wireless network capacity globally suggest that technical barriers to adoption will be minimal, representing an unparalleled opportunity for the managed healthcare industry to transform itself [27, 28]. Herein we dissect the various DMB component systems, and, by incorporating contextual elements, offer a standardization framework which will inspire its rapid evolution.

## Evolution of Digital Sensors for Clinical Use

**Fig. 1:**
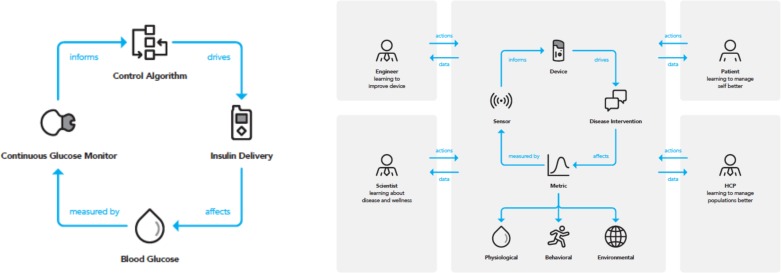
The closed-loop paradigm requires a concerted interdisciplinary approach to activate all domains. A traditional closed-loop system (left) can be used as part of a larger clinical feedback loop (right) that activates the 3 domains and different disciplines, allowing for a holistic and patient-specific approach to health and wellness management. HCP, healthcare provider.

The development of sensors for clinical use has been propelled by advances in the engineering of diagnostic and assessment tools. A digital sensor herein is defined as a device that employs an algorithm to measure physiological, biological, or cognitive information and provides feedback within a therapeutic area. The relative value of digital sensors reflects both the types of metrics that sensors can measure, and their adaptability to continuous monitoring of such metrics. In therapeutic settings, such continuous monitoring offers derived benefit with little to no intervention on the part of a clinician, caregiver, or patient, a prominent example being continuous glucose monitors for management of *diabetes mellitus* [29, 30]. The concept of a device containing a sensor that obtains a metric which provides insight and remedy for clinical decisions can be designed as of “closed-loop” embodiment. A prominent example in the diabetes area is the integrated system for glucose measurement and automated insulin delivery [31] which evolved in part due to pressure from patient-advocates [32] and has now been developed into a FDA-approved device, heralded as the “artificial pancreas” [33]. A priori, the closed-loop concept is considered domain-agnostic, focusing on a single metric or therapy. However, importing domains into the clinical feedback loop of health management allows a patient, technology company or healthcare provider holistic insight to an individual’s overall health. For example, environmental events included in this domain are external factors that contribute to stress, and by extension heart rate and cardiovascular health. A digital sensor with closed-loop capacity and also involving domain is a photoplethysmograph (PPG) whose sensor provides heart rate information [34]. The PPG sensors built into smartphones are not used to diagnose disease, so are therefore disease agnostic. However, a PPG sensor in a wristband can provide continuous heart rate monitoring and can then cross into the domain and diseased state, for example measuring difference in heart rate and blood volume changes which have been shown be used to diagnose peripheral arterial and vascular disease [35]. The power of these devices in cardiovascular health is evident. Though conventional gas chromatographic analysis measures analytes in serum to determine cholesterol levels that inform a physician to prescribe statins [36], it is appreciated that this is merely one metric obtained at one timepoint. A device with PPG sensors that provides information related to arterial disease means that the same information might ultimately be obtained without a blood draw and with the benefit of continual assessment. An additional benefit of a patient worn monitoring device could be to provide a time sensitive alert—for example, detection of the early onset of ischemic stroke. Similar applications of DMBs can be expected in Parkinson’s disease. No effective intervention is available for Parkinson’s disease and treatment for tremor control is only available after diagnosis. However, there is hope that the use of wearable devices that track heart rate and movement (with accelerometers) may provide relevant information before disease onset, allowing early intervention [37]. The embodiment of the clinical feedback loop model is depicted in Fig. 1, where the relationship between patient and clinician is augmented by devices, sensors, and metrics. A patient will be engaged with their physician (clinical) on the specific intervention or therapy prescribed to treat a condition. The patient may be deeply in tune with both the intervention and the device they must deploy or use to monitor a condition progression, but may be ambivalent to the specific sensors within the device or the metric that is being measured. The intersection of the sensor and the metric which it analyzes draws on a rich and evolving mix of science and engineering.

Physiological, environmental, and behavioral domains as well as disease categories stem from this closed-loop model. Implicit in the model is that a single disease state can have multiple domains which create or affect it, and likewise a single domain can be applicable to multiple disease areas. Application of such a technology driven model as a dashboard to inform healthcare provision is examined in the following section, with emphasis on amplified insight being provided by importing domains within the loop.

## Framework and Nomenclatures

**Fig. 2:**
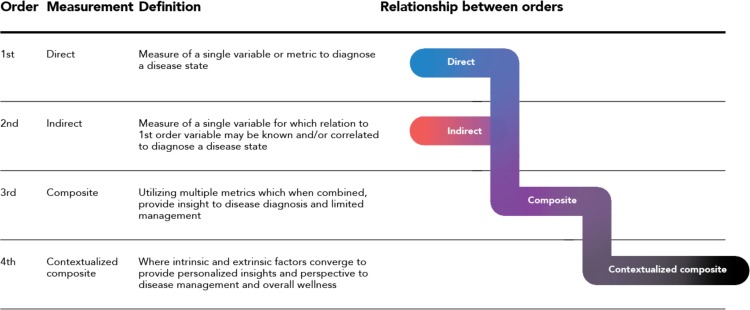
A composite biomarker value ladder progression from direct and indirect measurements (first and second order) where the physiological domain exclusively is considered, to a contextualize composite measurement (fourth order) comprised of physiological, environmental, and behavioral domains.

At the heart of the framework proposed here is the ideal that driving and increasing value for the patient must be the central focus of an expanded biomarker paradigm. The digital evolution of the past decades has enabled the shift, but ultimately the value must reside with the patient. Fig. 2 (and the associated Box) outlines an ordered approach to the evolution of a biomarker agnostic to therapeutic area.

Definition of first to fourth order: a first-order measurement is a direct measure of the disease or condition. It is the single metric used to either diagnose a state or quantify how a patient is managing their condition. Note that the simplicity or existence of a first-order metric can vary greatly between therapeutic areas. For example, a first-order measurement for diabetes is blood sugar level, an easily-obtained metric. This is compared to a number of neurodegenerative disorders where a detailed and complex brain synopsis is required postmortem.

A second-order measurement is an indirect measure of a state which has a known relation to the first-order metric. The second-order metric may often be used in place of the first order due to a variety of factors such as ease or simplicity of obtainment, cost, or intrusiveness. An example in the oncology space is in the detection of lung cancer, where a histological sample is obtained (first order) vs. a chest x-ray (second order). At large, the current framework of disease detection and monitoring is comprised of first and second order metrics. For both of these orders, the physiological domain associated with the different medical systems of the body is what drives measurements and diagnosis.

The digital progression has enabled a vision of where detection is heading via a third order, or composite biomarker, which is the combination of multiple metrics that provide insight to disease diagnosis and management. The increased value of a third order is demonstrated in therapeutic areas where a first and/or second order is either not known or not well defined. An example in the mental health space is the measurement of heart rate, sleep latency and heart rate variability, and their interaction to determine the onset or likelihood of an episode of depression. The composite biomarker is a paramount step towards self-identification and management, as multiple metrics that are simple and convenient for a subject to self-monitor and understand are combined into the composite biomarker without the burden or complexity of advanced diagnostic equipment only available solely in a clinical environment like a hospital or the physician’s office.

Ultimately, taking the third order composite biomarker and placing it in a subject’s surroundings creates a contextualized atmosphere. This is defined as the fourth order, where intrinsic and extrinsic factors are accounted for through behavioral and environmental parameters to provide ultimate insight into disease management. Consideration of an individual’s surroundings and their interaction with quantifiable data aids the understanding of how external factors affect that individual. As external factors play a pivotal role in one’s management of the disease, such as a patient’s emotional state or general feeling of well-being, bringing in this context is paramount to a complete management solution for subjects across all therapeutic areas.

The premise to the framework is a first- through fourth-order progression of metrics where the level of digital enablement increases directly with order. As a result of this progression, the framework highlights the state of simple diagnosis (first order) to a state where the patient is able to more effectively manage their condition or disease (fourth order). Note that while the outcomes of a first order biomarker are typically dependent on clinician intervention, the fourth order can be applied to a patient, clinician, or caregiver. In addition, as the orders progress from first to fourth, the behavioral and environmental domains augment the physiological domain.

**Fig. 3:**
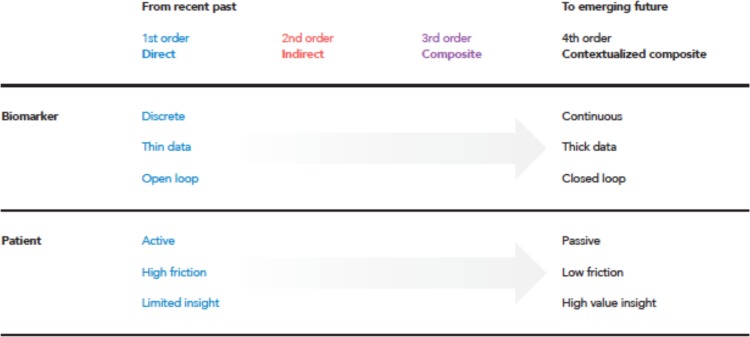
Moving from traditional first-order measures to diagnose disease, a trend of the recent past, to the future where both diagnosis and management is enabled through activation of contextualized composite metrics and simultaneously reducing the threshold of patient adoption, and reducing costs through economies of scale [38].

As the landscape described here moves across orders, there are 3 broader shifts that can be identified: digitally enabled data acquisition, a movement from diagnosing disease states to the management of these disease states by a patient directly, and an increase in the overall value to the patient. In a general categorization across therapeutic areas, Fig. 3 shows some of the characteristics of first order (direct measurements) versus fourth order (contextualized composite metrics).

Generally, first order measurements are discrete in time and obtained only at specific instances. These discrete measurements often produce thin data, or data which is managed via a single variable at a time. The first order enables open-loop systems where the patient must play an active role, making decisions in their disease area such as dosing amounts or whether to seek more advanced therapy such as surgery. The first order can be viewed as high friction to the patient, ultimately providing limited insight in how to manage their disease. As the fourth order is approached, there exists a shift in many of these tenets. Data are acquired for multiple variables continuously over time, creating dense, longitudinal data sets. The patient interaction and burden is removed via closed-loop systems. Taking away these obstacles produce enhanced patient value because active interaction is no longer required, lowering the friction of living with and managing a disease.

## Examples in Therapeutic Areas

**Fig. 4:**
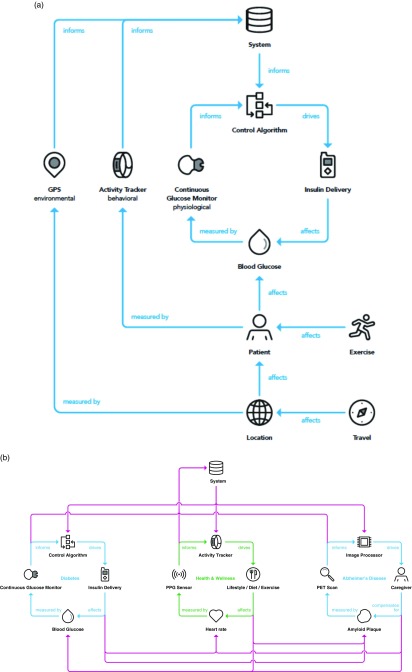
(*a*) Integrating the 3 domains (physiological, behavioral, and environmental) into the clinical feedback loop yields an opportunity to activate a contextualized composite metric that is relative to a specific person and provides optimized interventions at the right time. (*b*) A clinical feedback loop realizing the integration and interplay between comorbidities. An integrated system permits tailored interventions that are derived from the systematic and continuous interrogation of a person’s health and behavior. GPS, global positioning system; PET, positron emission tomography; PPG, photoplethysmogram.

A driving outcome of digital enablement of the contextualized composite biomarker is the ability to close the diagnosis and disease management loop while bringing in the domains inside the clinical feedback loop path. Ultimately, the value in a contextualized composite biomarker is the consideration of physiological and environmental factors, as demonstrated for diabetes in Fig. 4*a*. Instead of only monitoring glucose levels and controlling insulin delivery, behavioral aspects of diet, sleep [39], and exercise [40] are considered. As a patient understands the impact of sleep quality on prevention of hypoglycemic events, they are enabled to make decisions in their behavior to impact their glycemic control. In addition, accounting for environmental factors such as whether a patient is at home or traveling, and incidents in one’s personal life such as a death in the family or stress due to a sick child provide enhanced value to all aspects of a person’s life and care circle.

Although the most omnipresent embodiment for closed-loop care is in the diabetes space with the advent of the artificial pancreas for interactive glycemic control and insulin delivery, the framework described above could in principle be applied to any number of therapeutic areas. Fig. 4*b* demonstrates the application of the framework presented here to multiple disease states. Examples are provided of therapeutic areas where a closed-loop embodiment already exists (diabetes), a closed-loop architecture is dependent on a caregiver due to lack of an effective drug therapy (Alzheimer disease), and where a closed-loop embodiment is in place to prevent a therapy or intervention altogether (general wellness). Perhaps more compelling than application to diseases is to wellness and disease prevention, as the majority of US healthcare investment recently is focused on disease care rather than prevention based in part on reimbursement models [41].

As mentioned, the diabetes space is the most predominantly well known in the closed-loop space with the advent of the artificial pancreas both in academic research and commercial product development [31, 33]. A person with diabetes wears a continuous glucose monitor housing an electrochemical or optical sensor to acquire glucose measurements in the interstitial fluid (a second-order metric, of which a correlation to the first-order metric of blood glucose is established) [29, 30, 32]. Based on the glucose measurement, an insulin dosing recommendation is made and automatically delivered by an insulin pump [31, 33]. In the neurodegenerative domain, specifically for Alzheimer disease, a PET scan of the brain is conducted and using image processing techniques (the sensor), the amount of amyloid is quantified. A closed-loop paradigm (more aptly described as a *clinical feedback loop*) currently necessitates the intervention of a caregiver to make decisions for the patient, due to the lack of treatment or intervention available to eliminate this amyloid plaque build-up in the brain, which is one of the leading hypotheses for the onset of Alzheimer disease.

Moving to the more general area or human wellness, the contextualized composite biomarker provides the opportunity for a person to be continually informed and make decisions passively to enhance their overall well-being. A key difference with this application is that there is no therapy or intervention by a clinician, but rather daily decisions made by individuals such as how to eat, how much to exercise, as well as sleep decision and behavior, amongst others. These behaviors ultimately have the potential to keep people healthier while reserving the intervention of a clinician or practitioner to more advanced disease progressions or emergency situations.

**Fig. 5:**
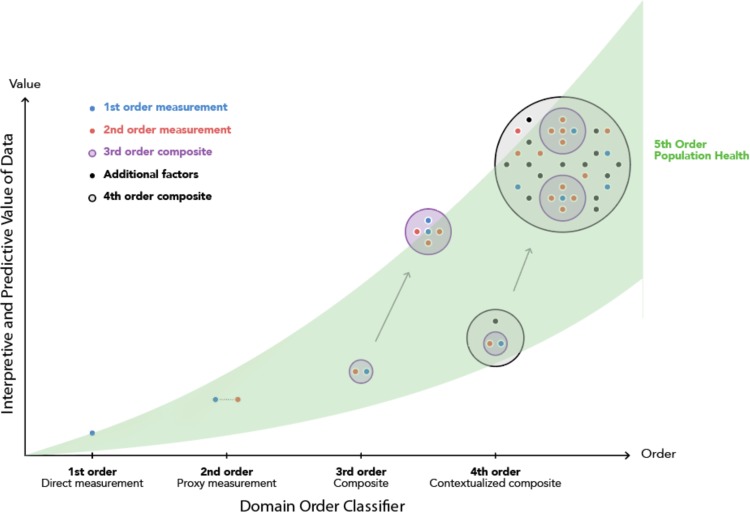
The cumulative and additive impact of domain order of composite monitoring biomarkers in clinical diagnostics and population health.

A potential extension from the contextualized composite biomarker would be to import this model to varying population segments and global health outcomes ubiquitously (Fig. 5). This extension to a “fifth order” would amplify the insight obtained for patients, clinicians, scientists, and engineers already achieved from embedding multiple domains inside the disease management model of the fourth order. In addition, it would enable activation in providing access to digital monitoring technologies irrespective of socioeconomic barriers [42].

## The Physicians Role in Applying the Principles

Though compelling in concept, the litmus test of a framework for use of contextualized DMBs in healthcare lies in its implementation. One possibility is the development of a digital dashboard for healthcare providers which is informed by these principles. For example, a diagnostic tool in the form of a “digital pinwheel” could be envisioned where various first- to fourth order parameters feed algorithmic decisions. Such a tool already has precedent, for example, from DBM-based studies of the impact of physical environment on risk for Lyme disease [43], and it is logical to consider how such devices could have been applied to monitor the spread of communicable diseases including SARS and avian influenza. Conversely, a *prognostic* digital pinwheel might also influence patient lifestyle and decision making, by rapidly assessing DBM’s based on composite metrics derived from physiologic, environmental, and behavioral inputs.

## Opportunities and Challenges

The emergence of ubiquitous, economical, and highly sensitive digital monitoring devices has the potential to play a major role in healthcare diagnosis of the future [24, 25, 34, 38, 43].

In tandem with these advances we have witnessed a paradigm shift in the adoption of many such tools into patients’ everyday lives, a consequence of the convenience of use of the devices and the widespread availability of broadband networks which they rely on. It is now imperative that the power of these systems is harnessed in broader elements of the managed healthcare ecosystem. It would seem timely for consumers who buy in to such a technology-centric approach. Recent data suggests that 14% of the US adult population are functionally illiterate (and, e.g., are unable to interpret prescribing information on drug packaging) [44] yet 68% own or have access to a smartphone [24, 45]. The emergence of voice assisted peripherals (Google Alexa, Apple Siri, etc) offers an opportunity to close this gap in the drug prescribing space. As advocated herein however, digital tools offer the opportunity to fully engage additional parties in the healthcare sector. Various studies underway are linking patient captured data to healthcare outcomes and it can be expected that a move from healthcare diagnosis and disease management will be augmented by strategies for wellness and disease prevention [46]. The adoption of these principles will require concerted and aligned effort between patients, providers, end-payors, and regulators. At the patient level, the term P4 medicine (predictive, preventative, personalized, and participatory) has been advanced, exemplified by longitudinal studies of large numbers of enrollees [47]. Numerous challenges will need to be addressed for mass implementation, including concerns on data privacy and security, and the adoption of uniform standards agreed by regulators [13]. It has been noted that considerable variation exists even with basic step counting technologies, and this will require standardization if classification as medical devices is desired [48]. Such data points incorporated as a component of a *composite* assessment, however, may allow a degree of self correction. Variability is also a concern with traditional clinical tests. An often cited example is the diagnosis of hyperthyroidism, which measures thyroid-stimulating hormone using an established range of 0.5–4.5 m IU/L [49]. Given variations in testing laboratory standardization, and the myriad other factors which are known to contribute to the reading, a composite approach to diagnosis is clearly preferable to a binary clinical decision based on a single (first order) metric [50]. As the field of DMBs evolves in managed healthcare it will impact all of its translational (T1–T4) components viz. translation to humans (T1), translation to patients (T2), translation to practice (T3), and translation to population health (T4). In the latter case, there is natural interest from end-payors and healthcare providers to lower costs and the very real potential to impact the insurance premium pools could exist. Many large organizations are now offering incentives for participants who enroll in wellness programs tied to monitoring biomarker trackers, and the actuary industry is likely to closely study these efforts for calculation of life insurance premiums [38]. The potential may exist for monitoring devices to provide instantly accessible diagnostic information at systems level, as is now customary in the automotive industry via the OBD-II port in a vehicle. Equally possible could be tracking devices which provide incentive to refrain from potentially detrimental behaviors (e.g., alcohol, tobacco consumption), which although logical prompts potential ethical and legal issues [13]. Medicine is conventionally taught, diagnosed, and treated on the basis of the major systems of the human anatomy (central nervous system, pulmonary, cardiovascular, muscular, skeletal, and gastrointestinal, etc). The availability of reliable, DMBs for each system would allow healthcare professionals deep insight to diseases, and help fully realize the vision for precision medicine [51].

## Conclusion

The widespread availability of digital devices capable of tracking monitoring biomarkers is poised to transform the managed healthcare sector. Advances in device design, sensor development, and cloud computing technology are expected to drive their ability to capture clinically relevant data sets. Already, devices with ability to interrogate and differentiate between normal and diseased states are facilitating design of insightful longitudinal patient centric studies which will highlight their clinical utility. Exploiting such enormous potential requires considered decision making at all levels of the healthcare system, and composite approaches offer considerable merit. We speculate that use of these technologies will readily augment conventional medical approaches, and in select therapeutic areas (CNS, neurodegenerative disorders) could provide ground breaking insight [52]. Equally importantly, use of such strategies to promote patient wellness and early warning of progression into diseased states has the potential to offer marked economic benefit to managed healthcare systems, and is already beginning to bear fruit [6]. To fully exploit the potential of digital medicine will also require the regulatory agencies to play a central role. Recent announcements from the FDA are encouraging, suggesting that digital health technologies will form a significant component of future guidance. For example the Digital Health Innovation Action Plan outlines numerous objectives, including a pilot precertification program for device developers [53]. Coupled with associated guidance documents this provides a framework for development of the digital biomarker industry [54], and the design of closed-loop devices [55]. Though the era of digital medicine is upon us, it is mindful to consider an oft-voiced quotation “A good decision is based on knowledge and not on numbers” (Plato). We would advocate that the key to modern medicine lies at the intersection of the two.
